# TSTA3 overexpression promotes malignant characteristics in LUSC by regulating LAMP2-mediated autophagy and tumor microenvironment

**DOI:** 10.1186/s12935-023-03109-z

**Published:** 2023-11-20

**Authors:** Yanlin Guo, Yanlong Hao, Liuyi Shen, Yu Du, Xiaohui Wang, Lvye Gao, Xuefei Feng, Yuanfang Zhai, Zhifei Liu, Enwei Xu, Yue Yang, Yanfeng Xi, Bin Yang, Ling Zhang

**Affiliations:** 1 Basic Medical Sciences Center of Shanxi Medical University, Taiyuan, Shanxi 030001 People’s Republic of China; 2 Fifth Middle School of Taiyuan, Taiyuan, Shanxi China; 3 Department of Pathology, Shanxi Cancer Hospital, Taiyuan, Shanxi China; 4 Department of Thoracic Surgery, Shanxi Cancer Hospital, Taiyuan, Shanxi 030001 People’s Republic of China

**Keywords:** NSCLC, LUSC, TSTA3, LAMP2, Lysosome

## Abstract

**Background:**

TSTA3 gene encoding GDP-l-fucose synthase has recently been proved to be closely related to the prognosis of patients with various tumors. However, its role in lung cancer is still unclear. The purpose of this study is to explore the expression level, prognostic effect, potential function and mechanism of TSTA3 in lung cancer.

**Methods:**

Based on TCGA database, Kaplan–Meier and COX regression was used to analyze the relationship between TSTA3 expression and prognosis of lung cancer patients. Immunohistochemistry was used to determine the TSTA3 protein expression in lung cancer and normal tissues. The function of TSTA3 in lung squamous cell carcinoma (LUSC) cell was determined by CCK8, colony formation, transwell assay in vitro and subcutaneous xenografts in vivo. Transcriptome analysis, Lyso-Tracker Red staining and rescue experiment were used to explore the possible underlying mechanism.

**Results:**

The expression of TSTA3 was significantly increased in lung cancer, especially in LUSC, and was significantly correlated with the malignant characteristics of LUSC. COX regression analysis showed that the high expression of TSTA3 was an independent prognostic factor in LUSC patients. This was also confirmed by immunohistochemical staining. Compared with the control group, the proliferation, colony formation, invasion and migration ability of LUSC cells with TSTA3 overexpression was enhanced. Similarly, the ability of cell proliferation, colony formation, invasion and migration were weakened after transient knockdown of TSTA3. In vivo experiment showed that compared with control group, TSTA3 overexpression significantly promoted the growth of tumor and shortened survival time. In addition, transcriptome sequencing analysis showed that the differentially expressed genes between TSTA3 overexpression and control group was mainly concentrated in the lysosome pathway. Further study found that TSTA3 might affect the proliferation, invasion and migration of LUSC by regulating the expression of lysosome-associated membrane protein 2 (LAMP2) in LUSC.

**Conclusion:**

The expression level of TSTA3 in LUSC is significantly higher than that in normal tissues. High expression of TSTA3 is associated with poor prognosis of LUSC patients. TSTA3 may affect the proliferation, invasion and migration of LUSC by regulating LAMP2.

**Supplementary Information:**

The online version contains supplementary material available at 10.1186/s12935-023-03109-z.

## Introduction

According to the latest global cancer burden statistics report, lung cancer has a high incidence rate and remains the leading cause of cancer-related death, causing about 1.8 million deaths (18%) worldwide [1]. In recent years, the treatment of cancer has been greatly improved, especially with the emergence of targeted therapy and immunotherapy, the survival rate of cancer patients has been greatly improved. However, it should not be ignored that this effectiveness only exists in 20% of cancer patients [2], and most cancer patients still cannot get very effective treatment. In lung cancer, for example, the effectiveness of treatment is closely related to molecular pathological and immune classification. Targeted therapy can be used for non-small cell lung cancer (NSCLC) patients with gene sensitive mutations, such as EGFR or ALK mutations, especially for those with lung adenocarcinoma (LUAD), and the survival rate is significantly better than that of patients without sensitive mutations. Similarly, the responsiveness of immunotherapy varies greatly in different patients and the effective response rate is relatively low [3]. Compared with LUAD, lung squamous cell carcinoma (LUSC) is less effective in targeting NSCLC and still lacks effective molecular targets for targeted therapy [4]. Therefore, it is essential to further explore the mechanism of pathogenesis and development of LUSC, search for novel biomarkers for early diagnosis, and develop new molecular targets for LUSC patients. Aberrant glycosylation is a hallmark of cancer which can impact many process in tumor progression from malignant transformation to distant metastasis and immune evasion [5]. Fucosylation is one of the most common glycosylation modifications and plays an important role in various cancers [6]. Fucosylated proteins have been recognized as promising biomarkers for malignancies, such as fucosylated alpha-fetoprotein for hepatocellular carcinoma, sialic Lewis a (sLe^a^, also known as CA19-9) for pancreatic cancer and CA 15-3 for breast cancer [7–9]. Furthermore, fucosylation study resulted in the identification of novel therapeutic targets for cancers. For example, specific fucosyltransferase inhibitors or neutralizing antibodies provided a new class of drugs for cancer therapy [10]. The l-fucose analogue 2-fluoro-l-fucose (2FF), which inhibits core fucosylation, results in reducing cell proliferation and migration in liver cancer cells [11]. Further studies of fucosylation in NSCLC are needed and may reveal new biomarkers for diagnosis and individualized therapy.

TSTA3 encodes GDP-l-fucose synthetase which is a key enzyme involved in fucosylation and is responsible for the production of GDP-l-fucose, the only donor of fucosylation [12]. It was found that TSTA3 harbored the significant mutation in liver cancer, which was closely related to the malignant characteristics of liver cancer cells. Inhibition of TSTA3 could decrease the migration and invasion of liver cancer cells significantly [13]. The activity of TSTA3 significantly affected the glycosylation process, and its abnormal mutations might lead to changes in fucosylation modification of eukaryotic proteins, thus affecting the structure and function of proteins [14]. In recent years, several studies have proved that TSTA3 was overexpressed, and involved in malignant progression and poor prognosis in gastric cancer, breast cancer and other cancers, which is involved in tumorigenesis and progression of tumors, and thus affecting the prognosis of patients [13–17]. Therefore, TSTA3 may serve as a novel tumor marker and potential therapeutic target for a variety of tumors. However, its expression, prognostic role and mechanism in lung cancer, especially in LUSC, are still unclear. This study aims to investigate the expression, prognostic value, potential function and possible mechanism of TSTA3 in NSCLC, and to provide theoretical basis for its use as a tumor marker and targeted therapy.

## Materials and methods

### Data processing

Data of TSTA3 mRNA expression in 482 cases of LUAD and 478 cases of LUSC and related clinical information were obtained from The Cancer Genome Atlas (TCGA) database (http://xena.ucsc.edu/public/). The data was used to compare the TSTA3 mRNA expression between lung adenocarcinoma, LUSC and normal adjacent tissues in TCGA database. The receiver operating characteristic curve (ROC) was applied to determinate the cut-off values of mRNA levels, divided the patients into high expression group and low expression group. The relationship between TSTA3 expression and clinical characteristics of patients was analyzed by SPSS. KM-plot analysis was used to reveal the influence of TSTA3 expression on the survival of LUSC and LUAD. COX regression analysis was used to determine the prognostic value of TSTA3 in NSCLC patients.

### Clinical samples

112 primary NSCLC tumor tissues (54 LUAD and 58 LUSC) and 66 matched normal lung tissues were collected from patients in the Shanxi Cancer Hospital (Taiyuan, China). All patients did not receive therapy before surgery, and normal tissues were collected at least 2 cm away from the tumor area in the same patients. The clinical data of each patient were complete, including age, sex, operative time, operation site and relevant pathological characteristic. The clinical outcomes of patients were obtained through telephone follow-up or outpatient visits. This study was approved by the Ethics Committee of Shanxi Cancer Hospital (Ethical batch number: 201845). The detailed clinical pathological information of the collected samples is exhibited in Additional file 1: Table S1.

### Cell culture

Human LUSC cell NCI-H226 (Cat#CL-0396) and NCI-H1703 (Cat#CL-0390) were purchased from the Procell Life Science&Technology (Wuhan, China). All cells were identified by STR, and STR typing showed no cross contamination of human cells in the cell lines. No mycoplasma was detected in the all cells. Cells were cultured in RPMI 1640 (Gibco, China, Cat#C11875500BT) containing 10% fetal bovine serum (FBS) (CellMax, China, Cat#SA211.02) and the culture dishes (Biofil, China, Cat#TCD010100) were placed in an incubator containing 5% CO_2_ at a constant temperature of 37℃. After the cells were overgrown in the culture dishes, the cells were digested and subcultured with 0.25% trypsin digestion solution (Meilunbio, China, Cat#MA0110).

### Immunohistochemistry (IHC)

The paraffin embedded and formalin fixed tissue microarray consisted of 112 primary NSCLC tumor tissues (54 LUAD and 58 LUSC) and 66 matched normal lung tissues was used. A total of 112 NSCLC patients were collected from Shanxi Cancer Hospital (Taiyuan, China). Histological classification and determination of metastasis were independently assessed by two senior pathologists.

The sections for IHC were deparaffinized and rehydrated with xylene and gradient alcohol. Then the sections were incubated with 3% H_2_O_2_ for 10 min to block the activity of endogenous peroxidase, and were blocked with goat serum at 37 °C for 20 min. The primary antibody TSTA3 (Abcam, Cat#ab190002) was diluted according to the ratio of 1:250 and incubated at 4 °C overnight. The secondary antibody (ZSGB-BIO, Cat#SP-9001) was incubated at 37 °C for 30 min. DAB kit (ZSGB-BIO, Cat#ZLI-9018) was used for staining. The slices were counterstained with hematoxylin, dehydrated with gradient alcohol, and covered with neutral gum. The stained specimens were scanned with an automatic digital section scanner (NanoZoomer SQ). Installed the plug-in IHC profiler in Image J, analyzed the slices and calculated the histochemical score (H-score). The receiver operating characteristic curve (ROC) of H-score was drawn by SPSS26.0 software to determine the optimum cut-off value.

### Plasmid constructs and transfection

For TSTA3 overexpression, plasmid constructs and lentiviral packaging were performed in OBiO Technology (Shanghai) Corp, Ltd. The CDS of TSTA3 gene was cloned into pLenti-CBh-GFUS-3xFLAG-P2A-Luc2-tCMV-mNeonGreen-F2A-Puro-WPRE and the empty vector was used as a control. The LUSC cell lines were inoculated into 24 well plates one day in advance. When the cell confluence reached 40%–50% on the second day, the diluted lentivirus diluent with different MOI gradients was added. After 20–24 h, the virus liquid was removed from the infected cells and 0.2 ml complete culture medium was added. Then, puromycin at a concentration of 2 μg/ml was used to screen stably overexpressing TSTA3 cell lines (designated as TSTA3 overexpression group, TSTA3-OE). Cells infected with negative control virus were classified as Con group. Subsequently, the 1 μg/ml of puromycin was used to maintain stable expression of TSTA3. Before the application of stably overexpression cells, western blot and qPCR was used to validate the overexpression of TSTA3. For LAMP2 overexpression, plasmid constructs were performed by WZ Biosciences Inc and the full length sequences was inserted into pCDNA3.1 (+)-P2A-GFP plasmid. For transfection, H226 cells were seeded at 60% density and Lipofectamine 2000 reagent (Thermo Fisher, Cat#11668-019) was used according to the manufacturer.

### TSTA3 knockdown

For knockdown of endogenous TSTA3, two siRNA sequences (Guangzhou RiboBio) were used: 5ʹ-CGGAGGCAGTTCATATACT-3ʹ (named as TSTA3-si1) and 5ʹ-CCGGAATATCAAATACAAT-3ʹ (named as TSTA3-si2). For transfection, H226 and H1703 cells were seeded at 50% density. siRNA transfection was performed using the Lipofectamine 2000 reagent (Thermo Fisher, Cat#11668-019) according to the manufacturer.

### Western blot and qPCR

RIPA protein lysate (Beyotime, Cat#P0013B) containing phosphatase inhibitor (Roche, Cat#04906845001) and protease inhibitor (Roche, Cat#11697498001) was used to lyse cells. After determining the protein concentration with BCA protein concentration determination kit (SEVEN, Cat#SW101-02), the extracted protein samples were separated by SDS-PAGE and transferred to PVDF membrane. The first antibody usage was as follows: TSTA3, Abcam, 1:1000; LAMP2, Abcam, 1:1000; Tubulin, Abmart, 1:4000; GAPDH, Immunoway, 1:6000. The horseradish peroxidase labeled secondary antibody (Immunoway, 1:10,000) was incubated at room temperature for 1–2 h, and then the ChemiDoc imager was used to detect chemiluminescence.

For qPCR, the total RNA was extracted from the cell precipitation using the RNA rapid extraction kit (Mei5bio, Cat#MF036-01). Reverse transcription to cDNA using the GoScriptTM reverse transcription kit (Promega, Cat#A5000). Relative gene expression was quantified using the comparative threshold cycle (2^−ΔΔCt^) method. GAPDH was used as internal control. The primer sequences used in this study are summarized in Additional file 1: Table S2. All experimental groups were provided with three duplicates.

### CCK8 and colony formation assay

For CCK8 assay, we seeded 3 × 10^3^ cells into each well of a 96-well plate in a final volume of 100 μl conditioned media. The plate was then placed into a cell incubator for 1–5 days. 10 μl of CCK-8 solution was added to each well and the plate was put in incubator at 37 °C for 4 h. The absorbance was measured at a wavelength of 450 nm using a microplate reader. For the colony formation assay, cells were seeded at a density of 1000 cells/well in a 6-well plate and incubated under standard conditions (37 °C, 5%CO_2_) for 14 days. After incubation, the cells were fixed with 4% polyformaldehyde for 20 min and dyed with 0.1% crystal violet for 20 min. Colonies consisting of more than 50 cells were subsequently counted using a microscope.

### Invasion and migration assay

For the invasion assay, the upper chambers of the transwell plates were pre-coated with 50 μl of BD Matrigel (1:6 mixed with FBS-free media; BD Biosciences, Germany, Cat#356234). Subsequently, 3–5 × 10^4^ cells were seeded into each well of the upper compartment using serum-free medium. The bottom of the chamber was filled with a medium that contained 10% FBS. For the migration assays, BD Matrigel was not utilized. After being cultured for 48 h, the cells on the upper surface were removed, and the cells that had passed through the membrane were fixed with 4% formaldehyde and stained with 0.1% crystal violet for 20 min. The five random fields were selected for cell counting.

### RNA sequencing

The transcriptome sequencing was completed by Beijing Genomics institution (BGI, Shenzhen, China). The sequencing raw data was filtered, afterwards clean reads were obtained and stored in FASTQ format. The subsequent analysis and data mining were performed on Dr. Tom II network platform (https://biosys.bgi.com). We conducted differential expression analysis using DESeq2 (v1.4.5) with a significance threshold of Q value ≤ 0.05. To gain insight into the phenotypic changes, we performed KEGG (https://www.kegg.jp/) enrichment analysis on the differentially expressed genes using the annotation. Significance levels of terms and pathways were adjusted using a rigorous threshold of Q value ≤ 0.05.

### Lyso-tracker red staining

For lysosomal staining, we mixed Lyso-Tracker Red (LTR) (Beyotime, Cat#C1046) with medium in a ratio of 1:16,000. The cells were seeded in six-well plates at the same density. LTR was added to six well plates and the cells were incubated in 37 °C for 40 min. Hoechst (Beyotime, Cat#C1027) was added to stain cell nucleus and the cells were washed two times with PBS, then we took photos under the fluorescence microscope.

### In vivo experiments

Female BALB/c-nude and NOD/SCID mice were obtained from Gempharmatech Co., Ltd in Jiangsu, China. The animal experiment received approval from The Ethics Committee of Shanxi Medical University and strictly followed the guidelines for tumor induction in mice. The mice were housed under standard, pathogen-free conditions, with a 12-h light/dark cycle and a temperature range of 22–26 ℃ and humidity between 45–65%. They were provided with free access to food and water. To assess the impact of TSTA3 on tumor growth in vivo, a mouse xenograft assay was performed. Each NOD/SCID mouse was subcutaneously injected with 3 × 10^6^ negative control or stable overexpressing TSTA3 of H226 cells into the left or right axilla. Tumor size was measured at regular intervals. After 4 weeks, tumors were removed and weighed. Moreover, metastatic tumor model was used to assess the impact of TSTA3 on tumor metastasis in vivo. Negative control and stable overexpressing TSTA3 of H226 cells (3 × 10^5^ cells in saline with a total volume of 175 μl) were injected into the tail vein of nude mice. After 55 days, the mice were euthanized by excessive injection of 2% sodium pentobarbital and the lungs and liver were dissected. The number of surface nodules of liver and lung was recorded and the tissues were stained with hematoxylin and eosin (HE) to verify the presence of tumor metastasis.

### Statistical analysis

The t-test was utilized to compare the differences between two groups, while univariate analysis of variance was employed to analyze the relationship between clinicopathological factors and prognosis. The statistical analysis was evaluated using GraphPad Prism 8 and SPSS 26.0 statistical software. The measurement data were expressed as mean ± standard deviation (Mean ± SD). The comparison between two groups was performed using the t-test, while the comparison between multiple groups was carried out using one-way ANOVA. P-value less than 0.05 were considered statistically significant.

## Results

### TSTA3 mRNA expression and its association with clinicopathological characteristics of NSCLC patients in TCGA database

**Fig. 1:**
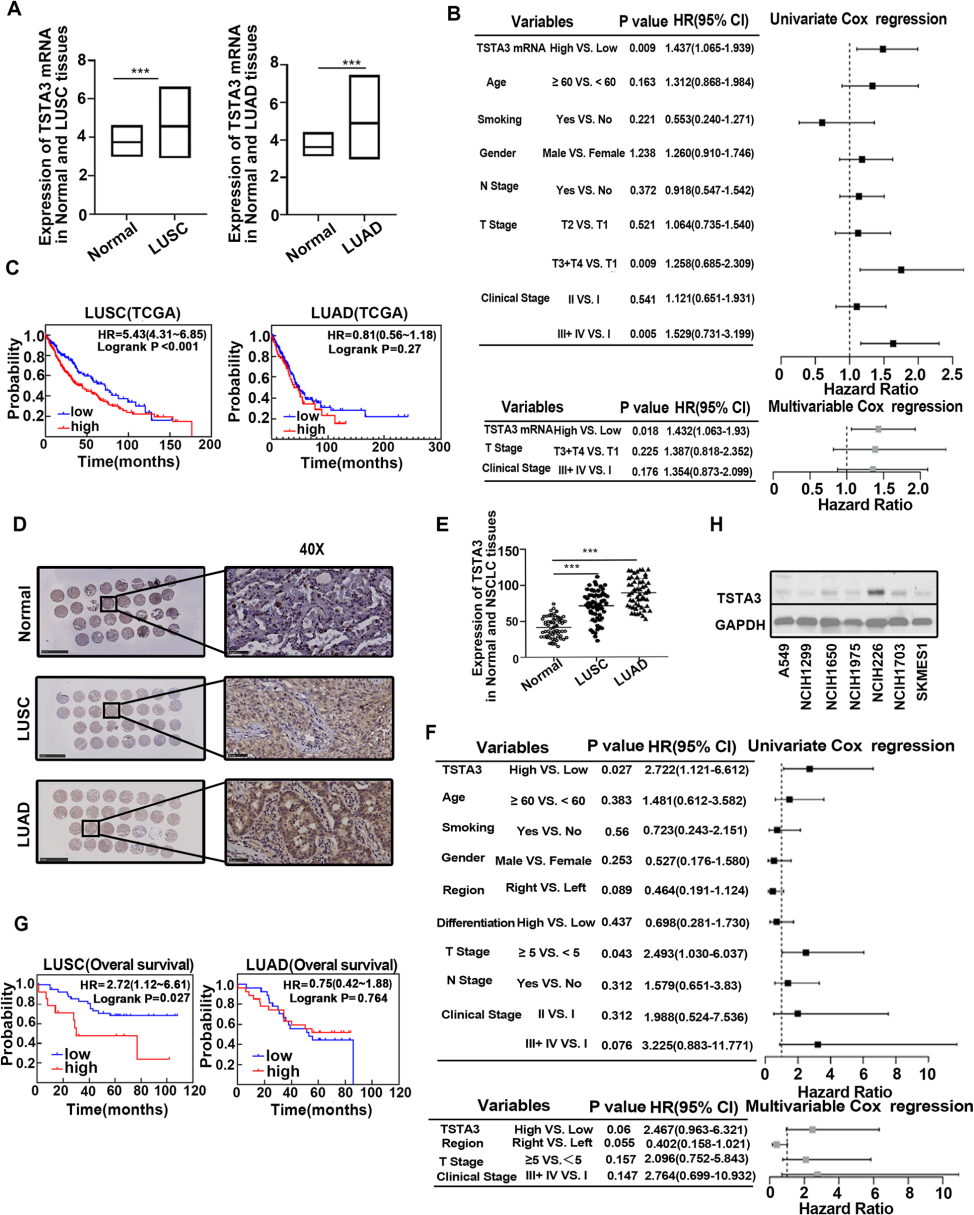
Patients with LUSC have elevated expression of TSTA3, which is a poor prognostic indicator. **A** TSTA3 mRNA expression in LUSC and LUAD based on TCGA Database (*** *P* < 0.001). **B** Univariate and multivariate COX regression analysis of LUSC patients based on TSTA3 mRNA expression in the TCGA Database. **C** Kaplan–Meier survival analysis of LUSC and LUAD patients in the TCGA Database. **D**, **E** Immunohistochemical experiments detected the location and levels of TSTA3 protein expression in normal tissues (n = 66), LUSC (n = 58), and LUAD (n = 54) tissues. Scale bar, 50 μm. **F** Univariate and multivariate COX regression analysis of LUSC patients based on TSTA3 protein expression detected by immunohistochemistry. **G** Kaplan–Meier analysis of LUSC patients and LUAD patients. **H** Western blot detected endogenous expression levels of TSTA3 in several NSCLC cell lines

As shown in Fig. 1A, the mRNA expression of TSTA3 in LUSC and LUAD were higher than that in normal tissues (*P* < 0.001). Based on the cutoff values determined by ROC curve, we divided the patients into high expression and low expression group to investigate the association between TSTA3 mRNA expression and clinicopathological characteristics. We found that TSTA3 expression was significantly associated with tumor size (*P* = 0.022) and clinical stages (*P* = 0.017) in LUSC patients (Table 1). Univariate Cox regression analysis showed that the expression of TSTA3 mRNA (*P* = 0.009), T stage (*P* = 0.009), and Clinical stage (*P* = 0.005) were significant prognostic factors for LUSC patients. After adjusting for potential confounders, multivariate Cox regression analysis showed that the high expression of TSTA3 was still an independent risk prognostic factor for LUSC patients (HR = 1.432, 95% CI 1.063–1.93, *P* = 0.018) (Fig. 1B). Moreover, Kaplan–Meier analysis showed that the LUSC patients with high expression of TSTA3 gene had a shorter survival time than those with low expression of TSTA3 (*P* < 0.001) (Fig. 1C). However, the expression of TSTA3 was not significantly associated with clinicopathological characteristics and prognosis in LUAD patients (Table 1, Fig. 1C and Additional file 2: Figure S1A).

**Table 1:** Association of TSTA3 mRNA levels with clinicopathological factors in patients with LUSC and LUAD in TCGA

Variables	Case	LUSC				Case	LUAD			
	n	Low (%)	High (%)	X^2^	P	n	Low (%)	High (%)	X^2^	P
Age (years)										
< 60	84	33 (19.5%)	51 (16.5%)	0.689	0.451	134	116 (28.1%)	18 (26.1%)	0.118	0.731
≥ 60	394	136 (80.5%)	258 (83.5%)			348	297 (71.9%)	51 (73.9%)		
Gender										
Female	126	37 (21.9%)	89 (28.8%)	2.687	0.105	263	230 (55.7%)	33 (47.8%)	1.475	0.225
Male	352	132 (78.1%)	220 (71.2%)			219	183 (44.3%)	36 (52.2%)		
Smoking history										
Never	18	5 (3.0%)	13 (4.2%)	0.470	0.619	264	229 (55.4%)	35 (50.7%)	0.532	0.466
Current	460	164 (97.0%)	296 (95.8%)			218	184 (44.6%)	34 (49.3%)		
T stage										
T1	106	39 (23.1%)	67 (21.7%)	7.653	0.022	165	148 (35.8%)	17 (24.6%)	4.073	0.130
T2	284	110 (65.1%)	174 (56.3%)			257	217 (52.5%)	40 (58.0%)		
T3 + T4	88	20 (11.8%)	68 (22.0%)			60	48 (11.6%)	12 (17.4%)		
N stage										
Yes	168	63 (62.7%)	105 (66%)	0.521	0.470	163	141 (34.1%)	22 (31.9%)	0.134	0.714
No	310	106 (37.3%)	204 (34%)			319	272 (65.9%)	47 (68.1%)		
Clinical stage										
I	236	84 (49.7%)	152 (49.2%)	8.197	0.017	261	222 (53.8%)	39 (56.5%)	2.918	0.232
II	153	64 (37.9%)	89 (28.8%)			120	108 (26.2%)	12 (17.4%)		
III + IV	89	21 (12.4%)	68 (22.0%)			79	83 (20.1%)	18 (26.1%)		

### Upregulation of TSTA3 in NSCLC tissues and as an independent risk factor for LUSC

Immunohistochemical staining based on tissue microarray showed that TSTA3 was mainly expressed in the cytoplasm (Fig. 1D). The H-score of TSTA3 expression in normal tissues, LUSC and LUAD tissues were 40.53 ± 14.39, 73.39 ± 20.91 and 88.91 ± 19.82 respectively. The expression of TSTA3 in LUSC and LUAD tissues was significantly higher than that in normal tissues (*P* < 0.01) (Fig. 1E). The cutoff values of TSTA3 expression in LUSC and LUAD tissues were determined by ROC curve and the patients were divided into high and low expression groups. Although the TSTA3 protein expression was not significantly associated with clinicopathological characteristics in LUSC and LUAD, univariate Cox regression analysis showed that the high expression of TSTA3 was significant prognostic factor for LUSC patients (Fig. 1F). Kaplan–Meier analysis also showed that the LUSC patients with high TSTA3 expression had a worse survival than those with low TSTA3 expression (*P* = 0.027) (Fig. 1G). No similar phenomenon was found in LUAD (Additional file 2: Figure S1B). We also detected endogenous expression levels of TSTA3 in several NSCLC cell lines by western blot. Consistently, as shown in the Fig. 1H, LUSC cells have relatively higher endogenous expression of TSTA3.

### Knockdown of TSTA3 inhibits migration, invasion and proliferation of LUSC cancer cells

**Fig. 2:**
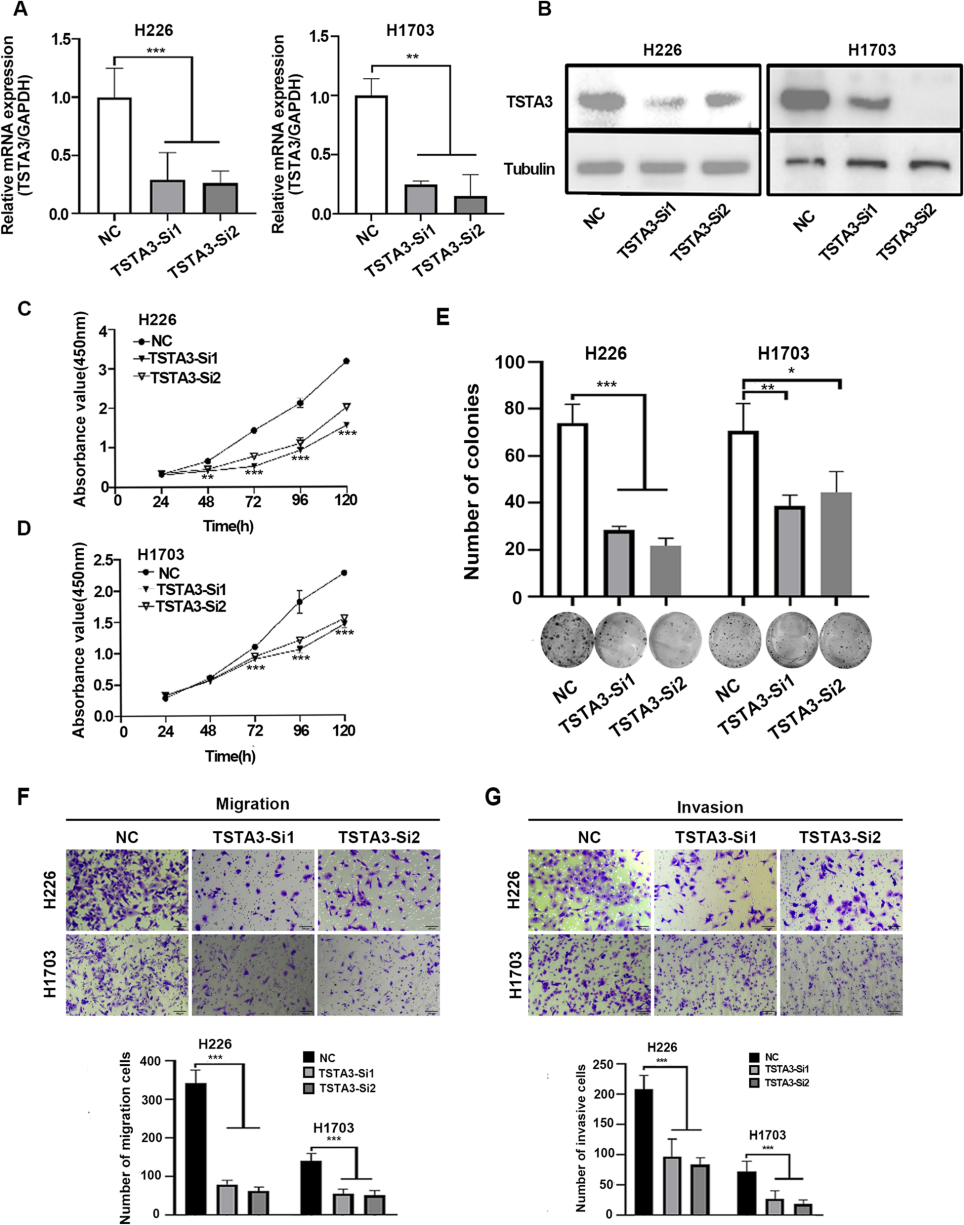
Knockdown of TSTA3 inhibits migration, invasion and proliferation of LUSC cancer cells. **A**, **B** RT-qPCR (**A**) and Western Blot (**B**) revealed that TSTA3 was efficiently knocked down in H226 and H1703 cells. **C**, **D** Analysis of the proliferation ability in TSTA3 knockdown H226 and H1703 cells by CCK8 assay. **E** Analysis of the proliferation ability in TSTA3 knockdown H226 and H1703 cells by colony formation assay. **F**, **G** Analysis of the ability of migration and invasion in TSTA3 knockdown H226 and H1703 cells by transwell assay. *** *P* < 0.001; ** *P* < 0.01; * *P* < 0.05

To further clarify the potential role of endogenous TSTA3 knockdown in LUSC cells, two siRNAs (TSTA3-si1, TSTA3-si2) targeting TSTA3 and a non-specific targeting siRNA (negative control, NC) were used. The efficiency of TSTA3 knockdown was validated by qPCR and western blot (Fig. 2A and B). CCK8 results showed that compared with NC group, the OD value of TSTA3 knockdown group in H226 cells decreased significantly at 48 h, 72 h, 96 h and 120 h (*P* < 0.01) (Fig. 2C), and notably reduced in H1703 cells at 72 h, 96 h and 120 h (*P* < 0.001) (Fig. 2D). In colony formation assay of H226 and H1703 cells, the number of colonies of TSTA3 knockdown group significantly decreased compared with NC group (*P* < 0.05) (Fig. 2E). TSTA3 knockdown significantly attenuated colony formation and proliferation ability of LUSC cells.

Next, we investigated the influence of TSTA3 knockdown on LUSC cell invasion and migration using transwell chamber coated with or without matrigel. As showed in Fig. 2F and G (*P* < 0.01), the number of migrated and invaded cells was significantly lower than that of NC group in both H226 and H1703 cells. TSTA3 knockdown markedly reduced the invasion and migration ability of LUSC cells.

### Overexpression of TSTA3 promotes migration, invasion and proliferation of LUSC cells in vitro and in vivo

**Fig. 3:**
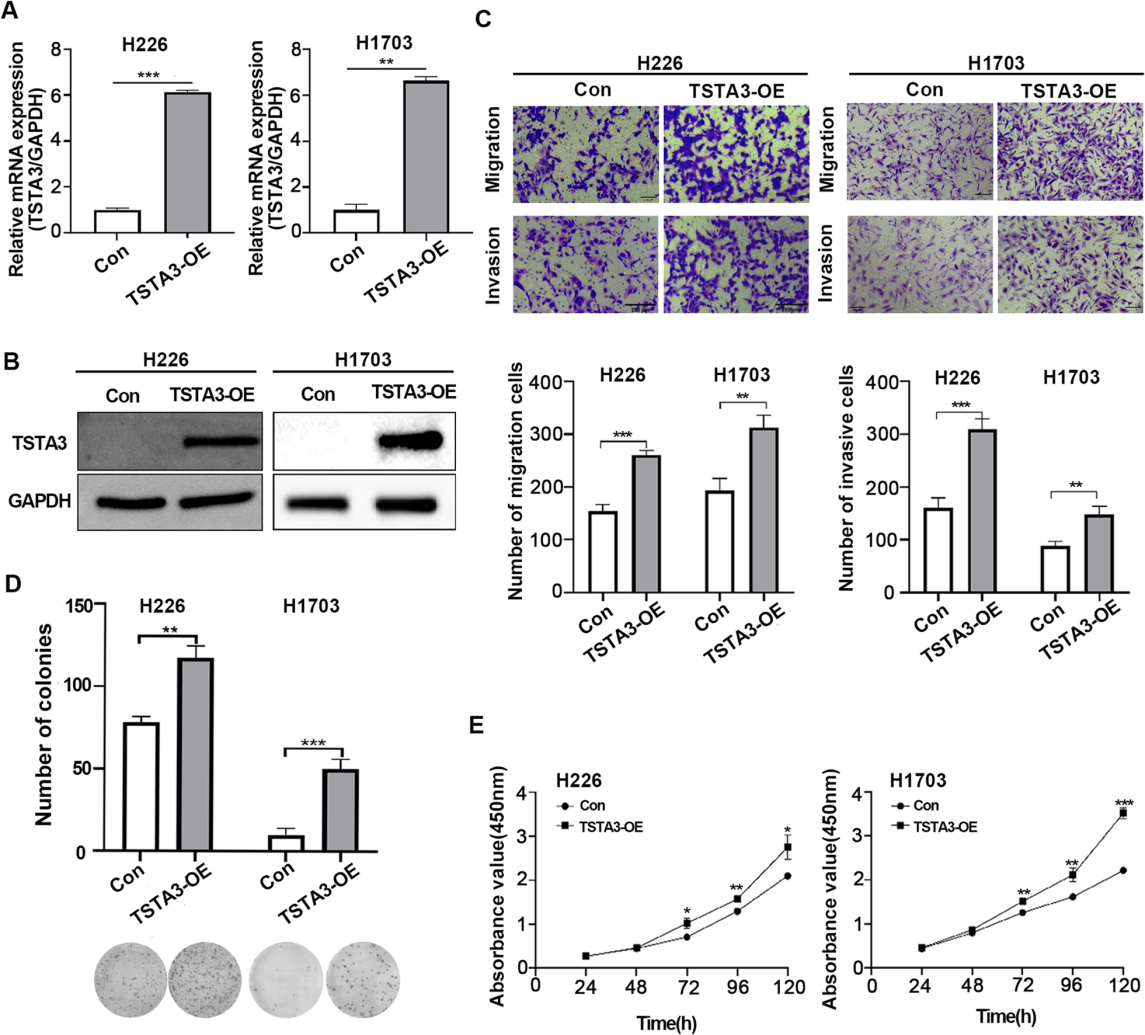
Overexpression of TSTA3 promotes migration, invasion and proliferation of LUSC cancer cells. **A**, **B** RT-qPCR (**A**) and Western Blot (**B**) revealed that TSTA3 was efficiently overexpressed in H226 and H1703 cells. **C** Analysis of the ability of migration and invasion in TSTA3 overexpression H226 and H1703 cells by transwell assay. Scale bar, 250 μm. **D**, **E** Analysis of the ability of proliferation in TSTA3 overexpression H226 and H1703 cells by colony formation assay (**D**) and CCK8 assay (**E**). *** *P* < 0.001; ** *P* < 0.01; * *P* < 0.05

To further reveal the function of TSTA3 in LUSC cell, H226 and H1703 cell lines with stably overexpressing TSTA3 gene (TSTA3-OE) were constructed. The efficiency of TSTA3 overexpression were detected by qPCR (Fig. 3A) and western blot (Fig. 3B). As shown in Fig. 3C, transwell assay showed that the migration and invasion ability of TSTA3-OE group were significantly enhanced compared with Control (Con) group, either H226 or H1703 cells (*P* < 0.01). Colony formation results demonstrated that the colony number of H226 and H1703 in TSTA3-OE group was much higher than that of Con group (*P* < 0.01) (Fig. 3D). Consistently, compared with Con group, the OD value of TSTA3-OE group in the two kinds of cells increased significantly at 72 h, 96 h and 120 h (*P* < 0.05) (Fig. 3E). In summary, TSTA3 exogenous overexpression significantly promoted cell migration, invasion, proliferation and colony formation of LUSC cells.

**Fig. 4:**
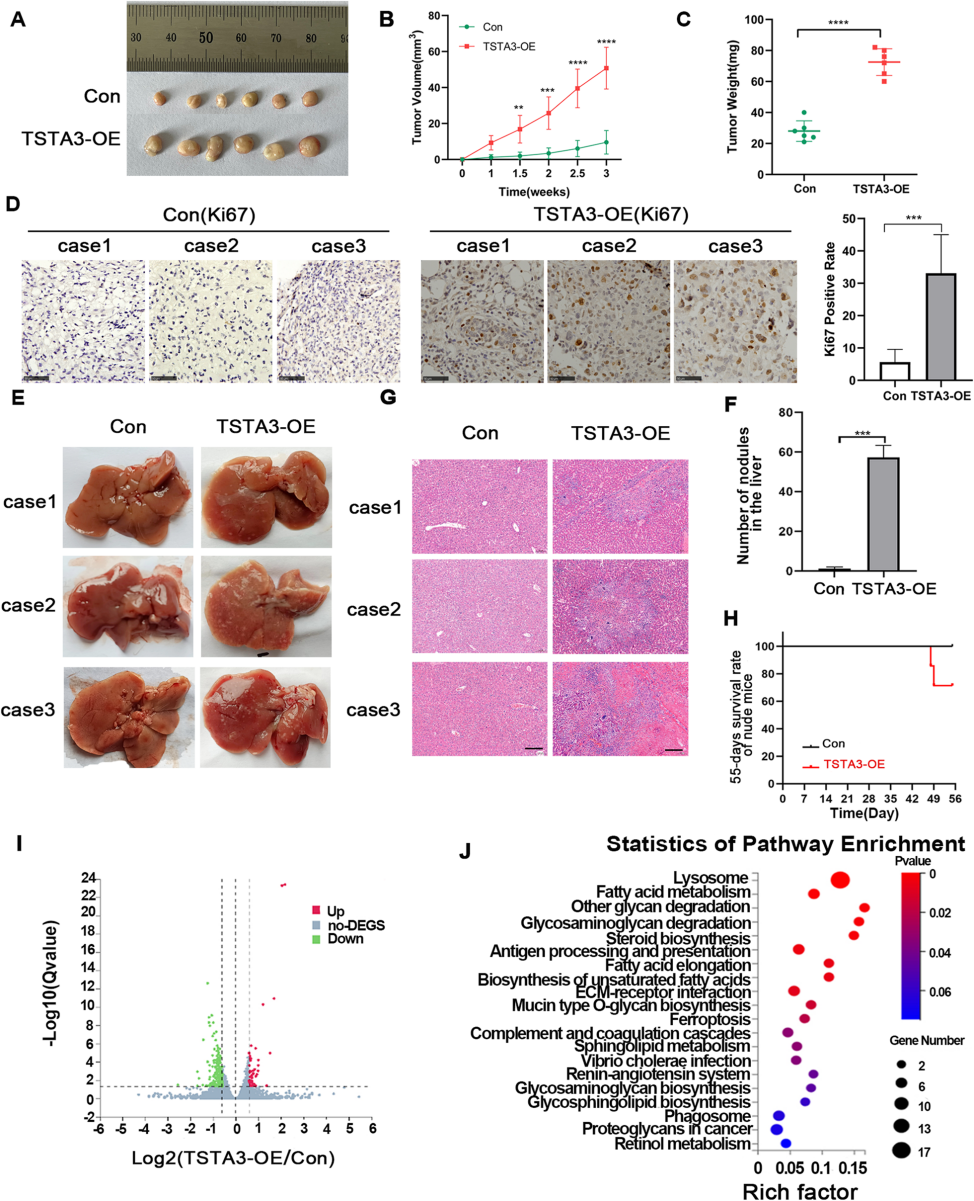
The effect of TSTA3 overexpression on the proliferative and metastatic ability of LUSC in vivo*.* **A** Tumor tissues in a subcutaneous tumor xenograft model (n = 6). **B**, **C** Tumor growth curves (**B**) and tumor weight (**C**) of subcutaneous tumor xenograft model (n = 6). **D** Immunohistochemistry detects the expression of Ki67 in xenograft tumor tissues of xenograft model mice. Scale bar, 50 μm. **E** Representative images of liver tissues of the NC group and TSTA3-OE group. **F** The number of liver metastatic nodules in NC group and TSTA3-OE group. **G** HE staining of liver tissues in NC group and TSTA3-OE group. Scale bar, 100 μm. **H** Survival curves of NC group and TSTA3-OE group. **I** Volcanic map of RNA sequencing in TSTA3 overexpression and negative control group H226 cells, with 47 genes up-regulated and 221 genes down-regulated in transcriptome data with fold change > 0.585 and *P* < 0.05. **J** Bubble diagram of KEGG-based enrichment analysis was performed for those 221 genes down-regulated with significant differences. **** *P* < 0.0001; *** *P* < 0.001; ** *P* < 0.01

To further determine the effect of TSTA3 overexpression on the proliferative and metastatic ability of LUSC in vivo, we firstly established a subcutaneous tumor xenograft model using H226 TSTA3-OE and control cells. The results showed that the tumor volume of TSTA3-OE group was significantly larger than the control group, and the tumor weight of TSTA3-OE group was also heavier than the control group (*P* < 0.001) (Fig. 4A–C). Furthermore, immunohistochemical staining of Ki-67 confirmed the growth promoting effect of TSTA3 overexpression in LUSC (Fig. 4D). The results indicated that TSTA3 might act as an oncogene in LUSC. Then we injected H226 cells stably overexpressing TSTA3 or empty vector into the tail vein of nude mice, and observed lung and liver metastasis. Although there were no visually significant nodules in lungs, the number of hepatic surface nodules in mice injected with H226 cells of TSTA3-OE was significantly higher than that in the control group (*P* < 0.001) (Fig. 4E and F). HE staining also confirmed that the number of liver nodules in TSTA3-OE group was significantly higher than that in control group, and there were more necrotic cells (Fig. 4G). Compared with the NC group, the TSTA3-OE group had poorer physical condition and shorter survival time (Fig. 4H). The above results showed that overexpression of TSTA3 in LUSC cells promoted the metastasis in vivo.

### Correlation between TSTA3 expression and lysosomal pathway in LUSC based on transcriptome data

**Fig. 5:**
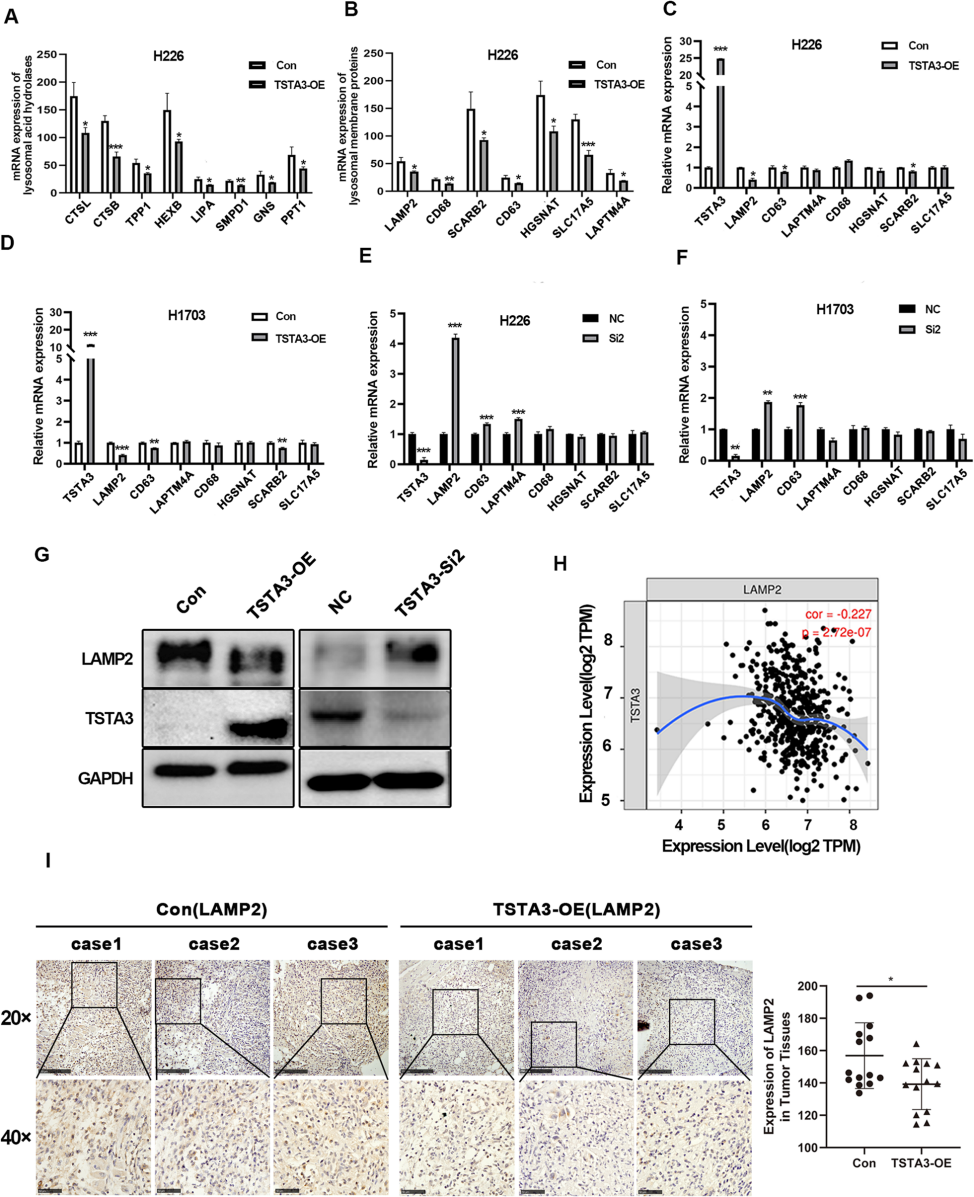
Correlation between TSTA3 expression and lysosomal pathway in LUSC. **A**, **B** The expression of lysosomal acid hydrolase (**A**) and lysosomal membrane proteins (**B**) in H226 cells with TSTA3-OE and NC group by RT-qPCR. **C**, **D** The mRNA expressions of LAMP2, CD63, LAPTM4A, CD68, HGSNAT, SCRAB2 and SLC17A5 in TSTA3 overexpressing H226 (**C**) and H1703 (**D**) cells. **E**, **F** The mRNA expressions of LAMP2, CD63, LAPTM4A, CD68, HGSNAT, SCRAB2 and SLC17A5 in TSTA3 knockdown H226 (**C**) and H1703 (**D**) cells. **G** The protein level of LAMP2 in TSTA3-knockdown or stably overexpressed H226 cells by Western blot. **H** Determine whether LAMP2 and TSTA3 are correlated using the TIMER database. **I** Immunohistochemistry detects the expression level of LAMP2 in tumor tissues of xenograft mice. Scale bar, 250 μm and 50 μm. *** *P* < 0.001; ** *P* < 0.01; * *P* < 0.05

In order to further clarify the downstream molecular mechanism of TSTA3 in LUSC, we performed RNA sequencing on TSTA3-OE H226 cells and control cells. Three independent replicates were conducted. The results revealed that compared with H226 NC group, 47 genes were significantly up-regulated and 221 genes were significantly down-regulated in TSTA3-OE group (|log2FC|≥ 0.585 and *P* < 0.05) (Fig. 4I. ). KEGG enrichment analysis showed that 47 up-regulated genes were mainly enriched in Apelin, Pentose phosphate, PI3K-Akt signal pathway, while 221 down-regulated genes were mainly enriched in lysosome, fatty acid metabolism, steroid biosynthesis, ferroptosis and other signaling pathways, of which lysosome pathway was the mostly significant (*P* < 0.05) (Fig. 4J). The lysosomal acid hydrolase (CTSL, CTSB, TPP1, HEXB, LIPA, SMPD1, GNS, and PPT1) and lysosomal membrane proteins (LAMP2, CD68, SCARB2, CD63, HGSNAT, SLC17A5, and LAPTM4A) were significantly down-regulated at the mRNA level in TSTA3-OE group compared with control group (Fig. 5A and B).

Using qPCR, the mRNA expressions of LAMP2, CD63, and SCRAB2 were verified to be downregulated in TSTA3 overexpressing H226 and H1703 cells (*P* < 0.05) (Fig. 5C and D). The mRNA expressions of LAMP2 and CD63 were verified to be upregulated in TSTA3 knockdown H226 and H1703 cells (*P* < 0.01) (Fig. 5E and F). Western blot validation of lysosomal membrane proteins showed that in H226 cells, the expression of LAMP2 protein decreased after overexpression of TSTA3, but increased after knockdown of TSTA3 (Fig. 5G). In addition, consistent with the sequencing results, the analysis results of TIMER database showed that LAMP2 expression had a negative correlation with TSTA3 (*P* < 0.001) (Fig. 5H). Moreover, we found the expression of LAMP2 was dramatically decreased in xenografted tumor tissue from mice injected with TSTA3 overexpressing LUSC cells compared with control cells (Fig. 5I).

**Fig. 6:**
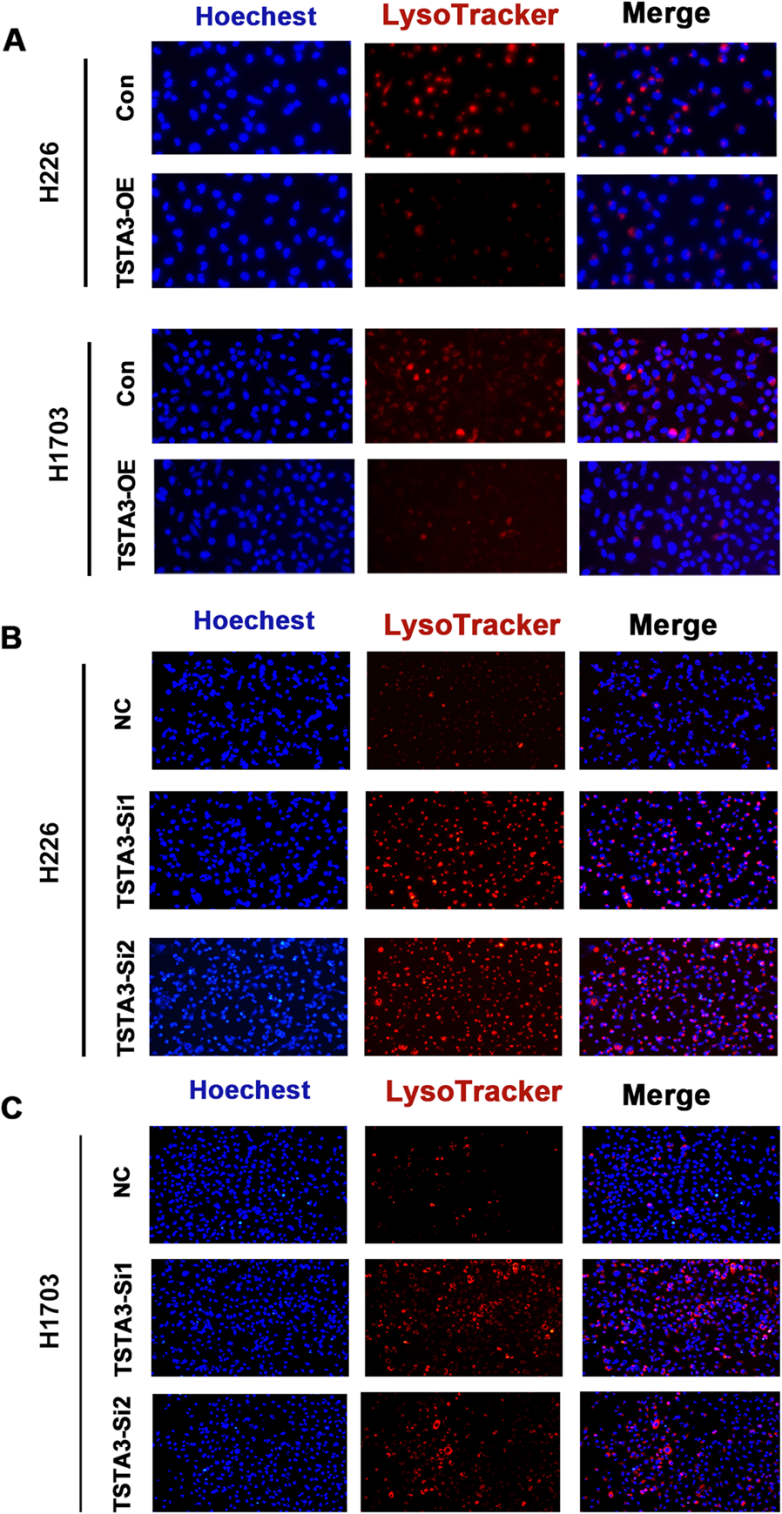
Lysosomes were tracked using the LTR probe to observe lysosomes changed with TSTA3 expression. **A** Fluorescence intensity and quantity of TSTA3 overexpressed H226 and H1703 cells. **B**, **C** Fluorescence intensity and quantity of TSTA3-knockdown H226 and H1703 cells

In order to further observe whether lysosomes change with TSTA3 expression, we used Lyso-Tracker Red (LTR) probe to trace lysosomes. The results showed that, compared with the control group, the fluorescence intensity and quantity were significantly decreased in H226 and H1703 cells with overexpressing TSTA3 (Fig. 6A), but significantly increased when TSTA3 was knocked down (Fig. 6B and C). LTR assay indicated that TSTA3 expression might affect the number and PH of lysosomes, leading to the change of lysosomal membrane permeability.

### Overexpression of LAMP2 reverses the effects of TSTA3 on invasion, migration and proliferation in LUSC

**Fig. 7:**
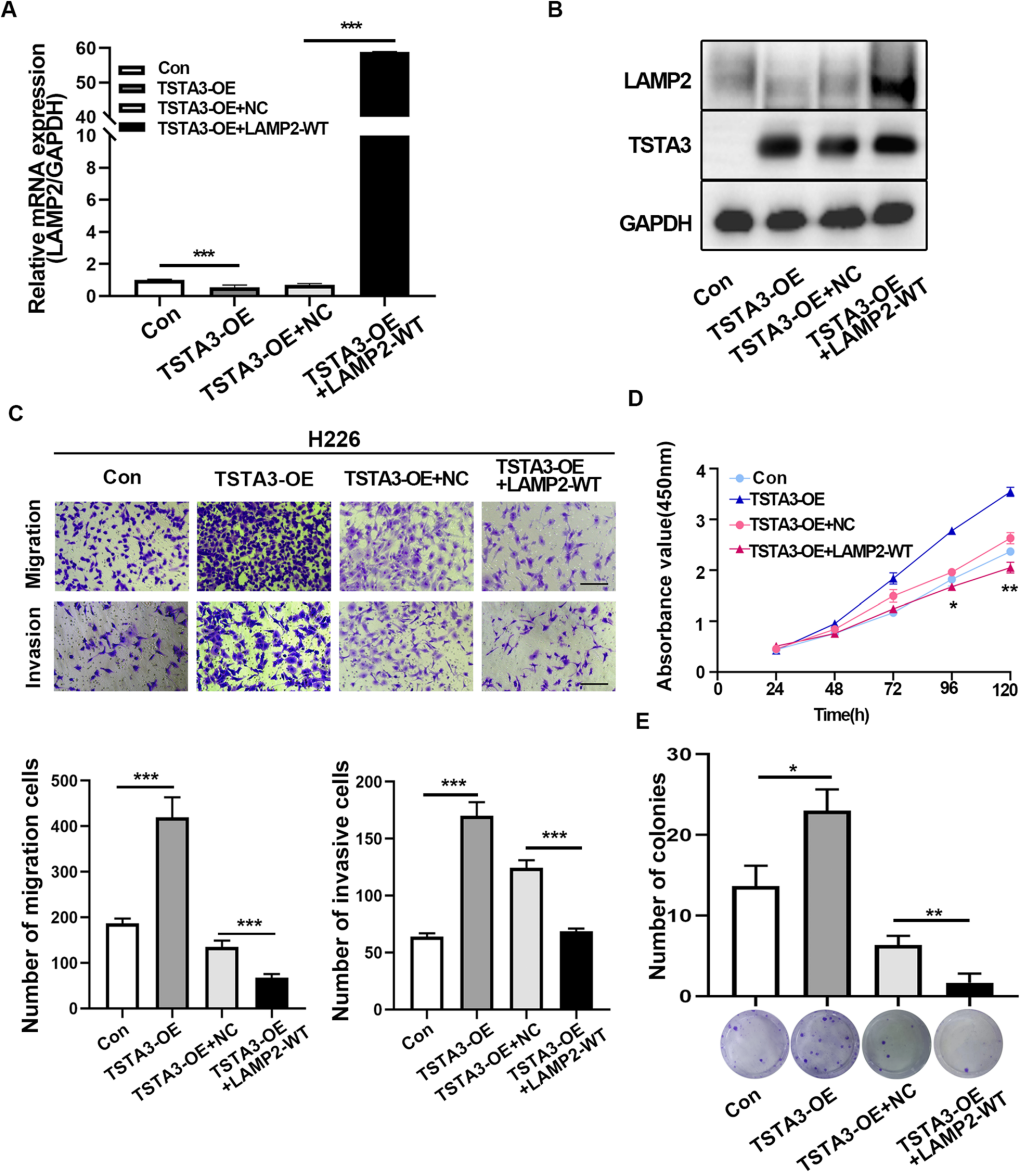
Overexpression of LAMP2 in LUSC cells reverses the impact of TSTA3 overexpression on malignant behavior. **A**, **B** The efficiency of LAMP2 overexpression in stable TSTA3 overexpressing H226 cells was determined by qPCR (**A**) and western blot (**B**). **C** Analysis of the ability of migration and invasion in stably TSTA3 overexpressed H226 cells with LAMP2 overexpression by transwell assay. Scale bar, 250 μm. **D**, **E** Analysis of the ability of proliferation in stably TSTA3 overexpressed H226 cells with LAMP2 overexpression by CCK8 assay (**D**) and colony formation assay (**E**). *** *P* < 0.001; ** *P* < 0.01; * *P* < 0.05

Previous studies found that changes in the expression of TSTA3 gene could stably regulate the expression of LAMP2 at mRNA and protein level, indicating that LAMP2 was a mediator of TSTA3 oncogenic effects in LUSC. LAMP2, known as lysosome-associated membrane protein 2, plays an important role in the protection, maintenance and adhesion of the lysosome. It was reported that LAMP2 also played a central role in tumor cell metastasis [18]. To further validate that LAMP2 may be a mediator of the oncogenic effect of TSTA3 in LUSC, we overexpressed LAMP2 in stably TSTA3 overexpressed H226 cells. The overexpression efficiency was validated by qPCR and western blot (Fig. 7A and B). As exhibited in Fig. 7C, overexpression of LAMP2 significantly attenuated the promotion effect of invasion and migration caused by TSTA3 overexpression in H226 cells (*P* < 0.001). Consistently, overexpression of LAMP2 in stably TSTA3 overexpressed LUSC cells had a significantly decreased proliferation ability compared with the control cells (*P* < 0.05) (Fig. 7D). Consistently, the increased capacity for the formation of colonies caused by TSTA3 overexpression in H226 cells was repressed by LAMP2 overexpression (*P* < 0.05) (Fig. 7E). All these results indicated that the phenotypes of TSTA3 overexpressed in LUSC cells could be reversed by LAMP2 overexpression.

## Discussion

TSTA3, also known as tissue specific transplantation antigen P35B, is a NADP(H)-binding protein and catalyzes the two-step epimerase and the reductase reactions in GDP-d-mannose metabolism. During this process, TSTA3 converts GDP-4-keto-6-d-deoxymannose to GDP-l-fucose [14]. GDP-l-fucose is a substrate for several fucosyltransferases and is involved in fucosylation of many glycoproteins [19]. Fucosylation is one of the common glycosylation modifications and abnormal fucosylation modification is closely related to the occurrence and development of cancers [20–23]. Evidence from research on colorectal cancer show that downregulation of TSTA3 expression could inhibit the colony formation, proliferation, migration and invasion ability of colorectal cancer cells and suppress the EMT process [24]. Our previous studies have shown that TSTA3 has significant copy amplification and overexpression in esophageal squamous cell carcinoma (ESCC) tissues, which was significantly related to the malignant progression and poor prognosis of ESCC patients [25, 26]. In addition, miR-125a-5p/TSTA3 axis also plays a role in breast cancer. Sun et al. showed that TSTA3 was highly expressed in breast cancer tissues and was closely associated with TNM stage. Overexpression of TSTA3 abnormally activated CXCR4/CXCL12 axis, thereby mediating cancer cell adhesion, metastasis and immune escape [16].

In this study, using TCGA database and microarray based on immunohistochemistry, we found that TSTA3 expression was significantly elevated in both LUSC and LUAD, but only indicated poor prognosis in LUSC patients. Functional experiments in vitro and in vivo demonstrated that TSTA3 promoted the proliferation, invasion and migration, and might act as a novel oncogene in LUSC progression. Further mechanism study using transcriptome sequencing, we found that the expression changes of TSTA3 could affect the expression of lysosomal related genes including the lysosomal acid hydrolase and lysosomal membrane proteins. The validation of RNA and protein levels in two LUSC cells confirmed that LAMP2 changes with TSTA3 expression. Moreover, LAMP2 overexpression could partially reverse the promotion effect of TSTA3 on proliferation, invasion and migration in LUSC cells.

Lysosomes, as intracellular degradation and signaling centers, play a crucial role in maintaining cell homeostasis, development, and aging by clearing protein aggregates, damaged organelles, and invasive pathogens [27]. It has been reported that lysosomes enhanced the malignant characteristics of cancer by altering their location, composition, and volume [28, 29]. Our study indicated that changes in the number and pH value of lysosomes caused by TSTA3 might participate in the malignant development of LUSC. Not only that, some studies have shown that cancer cells can release lysosomal enzymes (e.g. Cathepsin C, CatC) through exocytose to promote the degradation of the extracellular matrix, increase angiogenesis potential. At the same time, tumor derived CatC recruits neutrophils into metastatic foci through the CatC-PR3-IL-1β axis, thereby supporting the metastatic growth of tumor cells, and achieving local tumor progression [30]. The LAMP family is a lysosome-related glycosylated protein family that exists on the lysosome membrane. The family members mainly include LAMP1/CD107a, LAMP2/CD107b, LAMP3/DC-LAMP, LAMP4/CD68 and LAMP5/BAD-LAMP [31]. These members are expressed differently in different tissues, and their main functions focus on phagocytosis, autophagy, lipid transport and cell aging [32]. LAMP2 has three splicing isoforms, LAMP2A, LAMP2B, and LAMP2C. LAMP2A is considered to be responsible for molecular chaperone-mediated autophagy and acts on exosomes [33], LAMP2B are not associated with chaperone-mediated autophagy, but is involved in macro-autophagy. However, LAMP2C has been proved to be an inhibitor of molecular chaperone-mediated autophagy, especially in B cells, and can mediate nucleic acid autophagy by combining RNA and DNA [18, 34].

Several studies have shown that LAMP2 plays an important role in the development and progression of tumors. Jamali’s study showed that the expression of LAMP2 was significantly reduced in prostate cancer tissue and could trigger lysosomal membrane permeability, sensitizing cancer cells to lysosomal pathway mediated death. Compared to other autophagy related genes, LAMP2 was the best prognostic indicator and treatment target for prostate cancer patients [35]. Liu’s pan-cancer analysis of 25 common tumors revealed that LAMP2 significantly impacted the tumor microenvironment (TME) of immune infiltration, for example, LAMP2 was positively correlated with T central memory cells, Th2 cells and natural killer cell, but negatively correlated with Th17 cells [36]. Zheng’s study found that LAMP2 expression was significantly reduced in liver cancer and was correlated with metastasis, serum alpha-fetoprotein levels, vascular invasion, recurrence, and poor prognosis of liver cancer [37]. This might be attributed to LAMP2 associated epithelial-mesenchymal transition (EMT), as LAMP2 could inhibit the expression of Snail, upregulate E-cadherin, and inhibit EMT of liver cancer cells. Similarly, low expression of LAMP2 was associated with the malignancy of lung cancer. In lung cancer, miR-487b-5p downregulated LAMP2 protein, affecting their autophagy regulation and leading to drug resistance. LAMP2 could serve as a new marker for the target therapy of lung cancer [38]. Coincidently, in hematological diseases such as myelodysplastic syndrome (MDS) and acute myeloid leukemia (AML), LAMP2 has also been strongly associated with drug resistance and prognosis. Robert et al. showed that LAMP2 is decreased to various degrees in both MDS and AML cells resistant to azacytidine (AZA). Lack of LAMP2 expression also leads to decreased levels of autophagy and accumulation of cell fate determining proteins such as BCL2L10 and MLLT11 / AF1Q, resulting in enhanced Aza resistance and worse clinical outcomes [39]. Taken together, these findings show that the expression of LAMP2 is significantly reduced in some cancers, and affects the cancer cell itself and the tumor microenvironment. In this study, we found that TSTA3 overexpression can down-regulate the expression of LAMP2, and promote the proliferation, migration, invasion, and other malignant characteristics of LUSC. However, the specific mechanism and downstream cellular biological changes have not been fully revealed and need further study in the future.

To sum up, this study revealed for the first time that TSTA3 is overexpressed in LUSC, which promotes the growth, invasion and migration of tumor tissues, and can be used as an independent prognostic factor for patients with LUSC. This process may be achieved by affecting the expression and function of lysosomal membrane-associated protein LAMP2, which can affect the autophagy process and tumor microenvironment. Importantly, our research results also provide a new direction for targeted treatment of LUSC patients.

### Supplementary Information

**Additional file 1: Table S1.** Clinicopathological information in patients with LUSC and LUAD. **Table S2.** The primer sequences used for q PCR.

**Additional file 2: Figure S1.** COX regression analysis of forest plots in LUAD patients. **(A)** COX regression analysis forest map of LUAD patients in TCGA database. **(B)** COX regression analysis forest map of LUAD patients in immunohistochemistry cohort.

## Data Availability

The datasets used and/or analyzed during the current study are available from the corresponding author on reasonable request.

## Abbreviations

2FF: l-fucose analogue 2-fluoro-l-fucose
AML: Acute myeloid leukemia
AZA: Azacytidine
Con: Control
EMT: Epithelial–mesenchymal transition
ESCC: Esophageal squamous cell carcinoma
FBS: Fetal bovine serum
H-score: Histochemical score
IHC: Immunohistochemistry
LTR: Lyso-Tracker Red
LUAD: Lung adenocarcinoma
LUSC: Lung squamous cell carcinoma
MDS: Myelodysplastic syndrome
NC: Negative control
NSCLC: Non-small cell lung cancer
ROC: Receiver operating characteristic curve
TCGA: The Cancer Genome Atlas
TME: Tumor microenvironment
