# Supplementary material for: TSTA3 overexpression promotes malignant characteristics in LUSC by regulating LAMP2-mediated autophagy and tumor microenvironment
Source: Cancer Cell Int. 2023 Nov 20;23:285. doi: 10.1186/s12935-023-03109-z (PMC10662648; doi:10.1186/s12935-023-03109-z)
Supplement: Supplementary file 1 — Additional file 1: Table S1. Clinicopathological information in patients with LUSC and LUAD. Table S2. The primer sequences used for q PCR. [file 12935_2023_3109_MOESM1_ESM.docx]

**TSTA3 Overexpression Promotes Malignant Characteristics in LUSC by Regulating LAMP2-Mediated Autophagy and Tumor Microenvironment**

# Table S1: Clinicopathological information in patients with LUSC and LUAD

| **Clinicopathologic features** | **Classify** | **Number of cases of LUSC patients** | **Number of cases of LUAD patients** |
| --- | --- | --- | --- |
| **Age(Years)** | <60 | 25 | 31 |
|  | ≥60 | 33 | 23 |
| **Gender** | Female | 7 | 25 |
|  | Male | 51 | 29 |
| **Smoking history** | Never | 8 | 28 |
|  | Current | 50 | 26 |
| **Region** | Left | 28 | 24 |
|  | Right | 30 | 30 |
| **Differentiation** | Low | 36 | 24 |
|  | High | 22 | 30 |
| **T stage** | <5cm | 44 | 51 |
|  | ≥5cm | 14 | 3 |
| **N Stage** | No | 39 | 5 |
|  | Yes | 19 | 49 |
| **Clinical stage** | I | 16 | 23 |
|  | II | 21 | 10 |
|  | III+IV | 21 | 21 |

# Table S2: the primer sequences used for q PCR

| Primer Name | Forward Primer Sequence (5' to 3') | Reverse Primer Sequence (5' to 3') |
| --- | --- | --- |
| TSTA3 | CGTCATCCATCTTGCTGCAA | AGGTCGTCTTGTCAGGGAAG |
| GAPDH | TGACTTCAACAGCGACACCCA | CACCCTGTTGCTGTAGCCAAA |
| LAMP2 | AAATGGCACAGTGAGCACAAATGAG | GTTGTAGTAGGAGATGGCACAGTGG |
| CD68 | GTTCATCCAACAAGCAACAGCACTG | CGGAGAGGGTGGAGGTGGTTC |
| SCRAB2 | CGCTGGGTGTGTTCTTTGGTTTG | CTTTCATCCGCTGTTCCCTCATCC |
| HGSNAT | TTACAAGGCTCGGACCAAAGACATC | TCAGAAACCTTCGTCAGAGCAACAG |
| SLC17A5 | TCCTTCGTATGCTGGTATCCTCCTG | GAACACGGTTTGCCATTCTCCAAC |
| LAPTM4A | CGTGCCGGAGATTGCTGTGTAC | AGGCAGGTAAGTAAGGAGGTGGTG |
