# Supplementary figures and images for: TSTA3 overexpression promotes malignant characteristics in LUSC by regulating LAMP2-mediated autophagy and tumor microenvironment
Source: Cancer Cell Int. 2023 Nov 20;23:285. doi: 10.1186/s12935-023-03109-z (PMC10662648; doi:10.1186/s12935-023-03109-z)

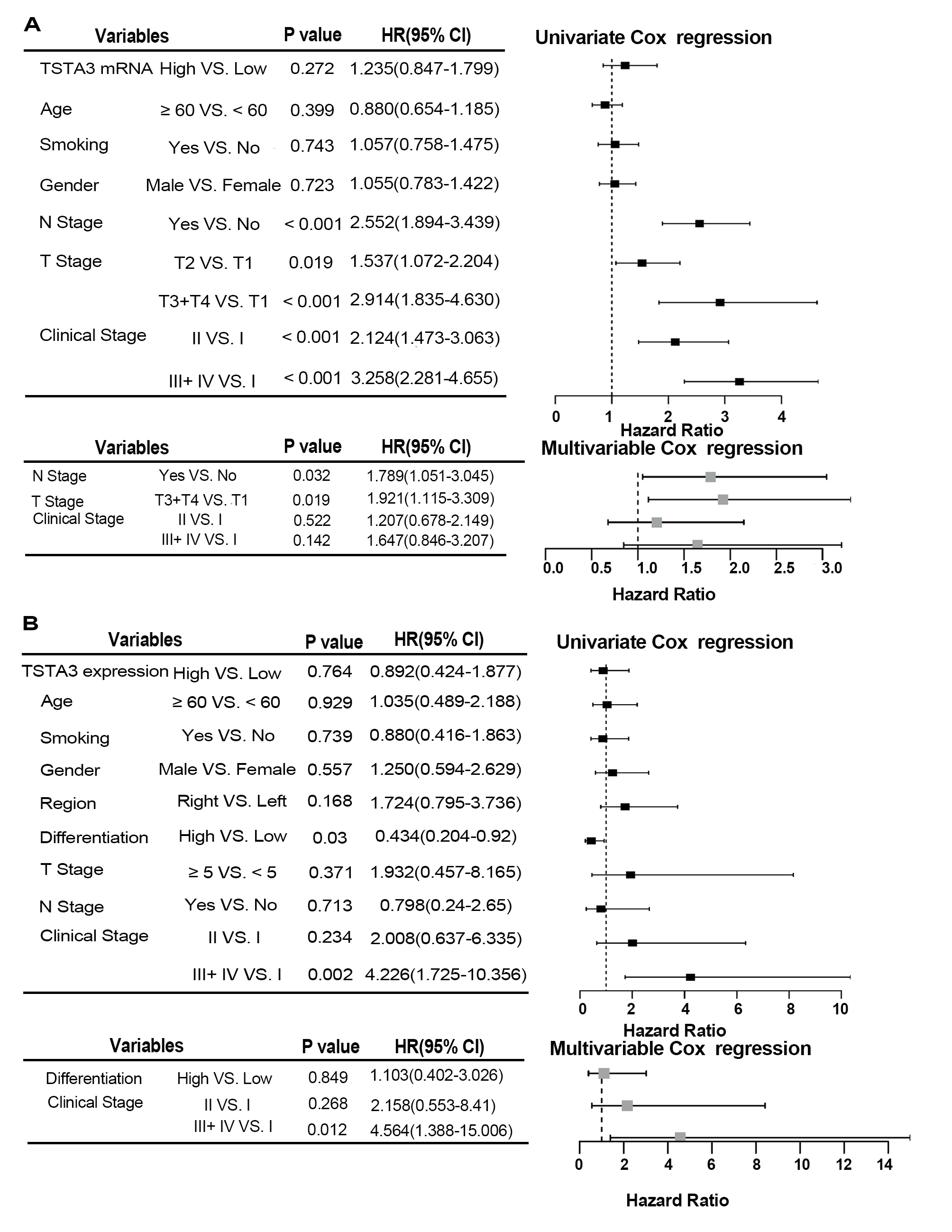

Supplement: Supplementary file 2 — Additional file 2: Figure S1. COX regression analysis of forest plots in LUAD patients. (A) COX regression analysis forest map of LUAD patients in TCGA database. (B) COX regression analysis forest map of LUAD patients in immunohistochemistry cohort. [file 12935_2023_3109_MOESM2_ESM.tif]
